# Basophil activation test as a biomarker in allergic patients to platins undergoing rapid desensitization

**DOI:** 10.1186/1939-4551-8-S1-A147

**Published:** 2015-04-08

**Authors:** Pedro Giavina-Bianchi, Matthieu Picard, Joana Caiado, Mariana Castells

**Affiliations:** 1 University of São Paulo, Brazil; 2 Brigham and Women's Hospital, Harvard Medical School, USA

## Background

Rapid Drug Desensitization (RDD) has become a cornerstone of the management of immediate hypersensitivity reactions (HSRs) to chemotherapeutic agents, including biologicals. It is the only effective procedure for overcoming HSRs to first-line therapy, thus representing an important advance in patients’ treatment and prognosis. Biomarkers to assess drug sensitization and monitor RDD safety and efficacy are lacking. Preliminary data suggested that in addition to skin testing, basophil activation test (BAT) could be used to assess patient’s IgE sensitization to platins and provide markers of activation to evaluate the response to RDD.

## Methods

We studied 12 patients with gastrointestinal and OBGYN cancers who presented hypersensitivity reactions to platins and 6 healthy volunteers who had never been exposed to platins. Skin testing and BAT were done before RDD to platins, and the expression of activation markers CD203c and CD63 was evaluated on basophils (HLADR-/CD41-/CD123+). Most patients were evaluated during 2 or more RDD procedures.

## Results

BAT was positive in 9/12 patients (75%), with increased expression of CD203c (9 patients) and CD63 (4 patients). The BAT positivity was 66,7% (6/9) for carboplatin and 100% (3/3) for oxaliplatin. Subsequent BAT analysis in different RDD procedures showed that the test remained positive before each procedure with an even greater expression of CD203c and CD63, indicating temporary tolerance during RDD, which was lost after each exposure. In an attempt to correlate reactions during RDD with specific BAT markers, we observed an association between CD63 expression and the severity of the reactions. All controls had negative tests. Further studies are needed to determine the predictive values of BAT in patients with platins hypersensitivity.

## Conclusions

We standardized a BAT to platins with good sensitivity and which can predict patients with severe reactions during RDD. RDD to platinum drugs does not induce persistent hyporresponsiveness on basophils, highlighting the need to maintain repeated RDD in allergic patients to platins.

